# Crystallization of NaCl–NaNO_3_–H_2_O in Porous Media During Drying

**DOI:** 10.1021/acs.cgd.4c01446

**Published:** 2025-04-09

**Authors:** Leo Pel, Sebastiaan Godts, Amelie Stahlbuhk, Noushine Shahidzadeh, Michael Steiger

**Affiliations:** † Department of Applied Physics, Transport in Permeable Media, Eindhoven University of Technology, P.O. Box 513, 5600 MB Eindhoven, The Netherlands; ‡ Monuments Lab, Royal Institute for Cultural Heritage (KIK-IRPA), 1000 Brussels, Belgium; § Department of Chemistry, University of Hamburg, Hamburg 20146, Germany; ∥ Van der Waals-Zeeman Institute, Institute of Physics, University of Amsterdam, Science Park 904, 1098 XH Amsterdam, The Netherlands

## Abstract

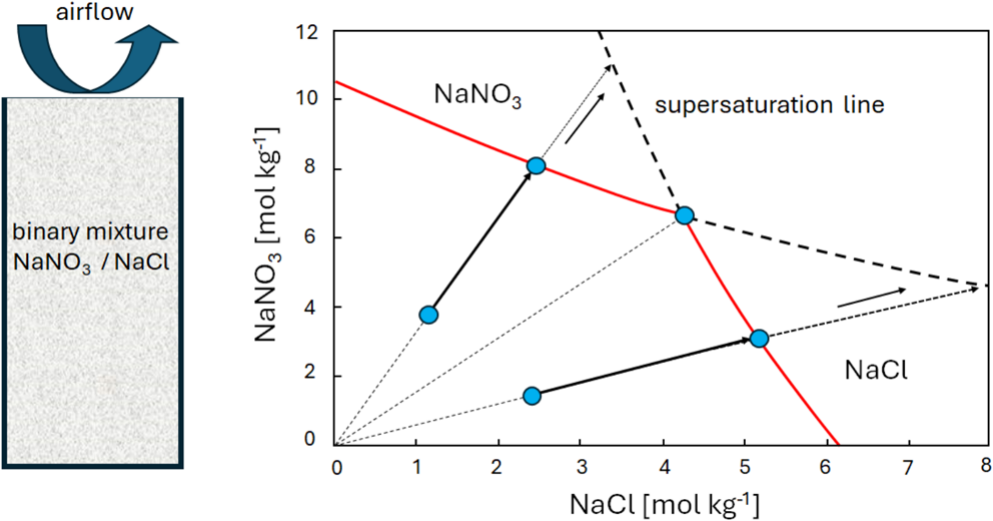

In this study, we investigated the crystallization of a salt mixture during drying in a porous medium. Specifically, we focused on the ternary system of NaCl–NaNO_3_–H_2_O, which is also encountered in situ. In order to study the crystallization, we use a specialized 4.7 T NMR setup that allows us to directly measure the NaCl and NaNO_3_ concentrations in a porous medium and track their ratio during drying, providing direct insight into the phase diagram. The measurements indicate that the equilibrium phase diagram alone is not sufficient to describe the physical processes that occur in porous media during drying experiments. In the case of forced drying in this study, where advection of the ions is dominant (*Pe* > 5), the measurements indicate that we need to take supersaturation into account and that crystallization is driven by transport. As a result, the ratio of a salt mixture will remain constant in the porous medium throughout the experiments, as was seen for this ternary system Na^+^, Cl^–^, NO_3_^–^ resulting in the formation of both NaCl and NaNO_3_. These results indicate that the rate of evaporation, in combination with the effect of supersaturation and solution transport in the pore system, allows the saturation degree given by the phase diagram to be surpassed. This phenomenon is critical when assessing mixed salt systems in porous media and should be considered when evaluating phase diagrams alone.

## Abstract 2

In this study, we investigated the crystallization of a salt mixture during drying in a porous medium. Specifically, we focused on the ternary system of NaCl−NaNO_3_−H_2_O, which is also encountered in situ.

## 1 Introduction

Salt crystallization processes play an important role in various damage mechanisms. In general, only the crystallization of pure salts is studied. However, salt mixtures are ubiquitous in our environment,^1^ and they intersect with a wide range of scientific fields where they are considered problematic due to their complex interactions, potential to disrupt natural processes, and the challenges they pose in various applications.^2^ Salt weathering of stone materials is of significant concern, as it leads to stone decay through pressures exerted by crystallizing salt within the pores and the formation of efflorescence on the surface. However, most studies focus on binary systems such as sodium (Na^+^) and chloride (Cl^–^).^3^ In contrast, realistic scenarios often involve at least five ions, including chloride (Cl^–^), nitrate (NO_3_^–^), sodium (Na^+^), potassium (K^+^), magnesium (Mg^2+^), sulfate (SO_4_^2–^), or calcium (Ca^2+^).^4^ Although theoretical models exist to understand the behavior of salt mixtures,^5^ these models typically assume equilibrium conditions and exclude kinetic considerations,^6,7^ such as supersaturation.^8−11^ Therefore, experimental investigations of salt mixtures are critical to comprehending the physical processes that occur. Both NaCl and NaNO_3_ are frequently identified in the built environment;^4^ thus, investigating the ternary system Na^+^, Cl^–^, NO_3_^–^ is considered a logical step forward.

**Figure 1:**
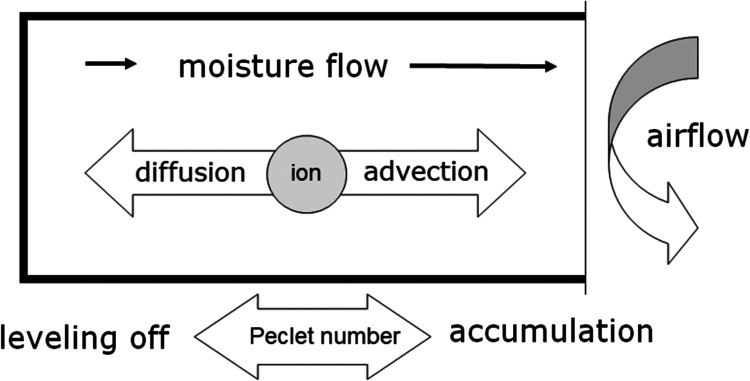
Schematic representation of the drying process in a porous medium saturated with a salt solution.

In this study, we focus on the crystallization of a salt mixture during drying in a porous medium, i.e., the crystallization under nonequilibrium conditions inside a porous medium. In this case, crystallization is driven by the drying process within the porous medium, as schematically shown in Figure 1. While the sample is drying, there will be a moisture flux toward the surface, and ions can be transported within the moisture. The ion transport itself can be described by a combined advection–diffusion equation. Here, the macroscopic diffusivity of the ions within a porous material is related to the microscopic diffusivity of the ions through the pores by the tortuosity. As a result, during drying, there will be competition between these two transport mechanisms, which can be characterized by a dimensionless number, i.e., the Peclet number (see, refs (12−14)).
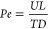
1where *U* is the liquid speed, *T* is the tortuosity, *D* is the microscopic diffusivity of the ion, and *L* is a characteristic length scale (which, in this case, can be chosen as the length of the sample). In the case of *Pe* > 1, advection dominates, leading to a buildup of ions and a concentration peak near the surface. As a result, crystallization predominantly occurs near the surface. Conversely, if *Pe* < 1, diffusion dominates, and a more homogeneous distribution of salt is expected.

For a salt mixture, the equilibrium constant for the dissolution reaction, known as the thermodynamic solubility product, is expressed as a function of the activity coefficients (γ*_M_* and γ*_X_*), the molalities of the cation *M* (*m*_*M*_), and anion *X* (*m*_*X*_) in the saturated solution.^14^
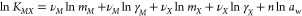
2where *n* is the stoichiometric coefficient for water (i.e., the number of water molecules involved per formula unit of salt) and *a*_w_ is the water activity.

It is evident from the given equation that the solubility of a salt is notably affected by the existence of a second salt that may share a common ion, consequently leading to an increase in either *m*_*M*_ or *m*_*X*_. Furthermore, it should be noted that the presence of a second salt, even though it does not share a common ion with the first salt, can still affect the equilibrium constant. This effect primarily occurs because the second salt modifies the activity coefficients of the ions *M* and *X.*

**Figure 2:**
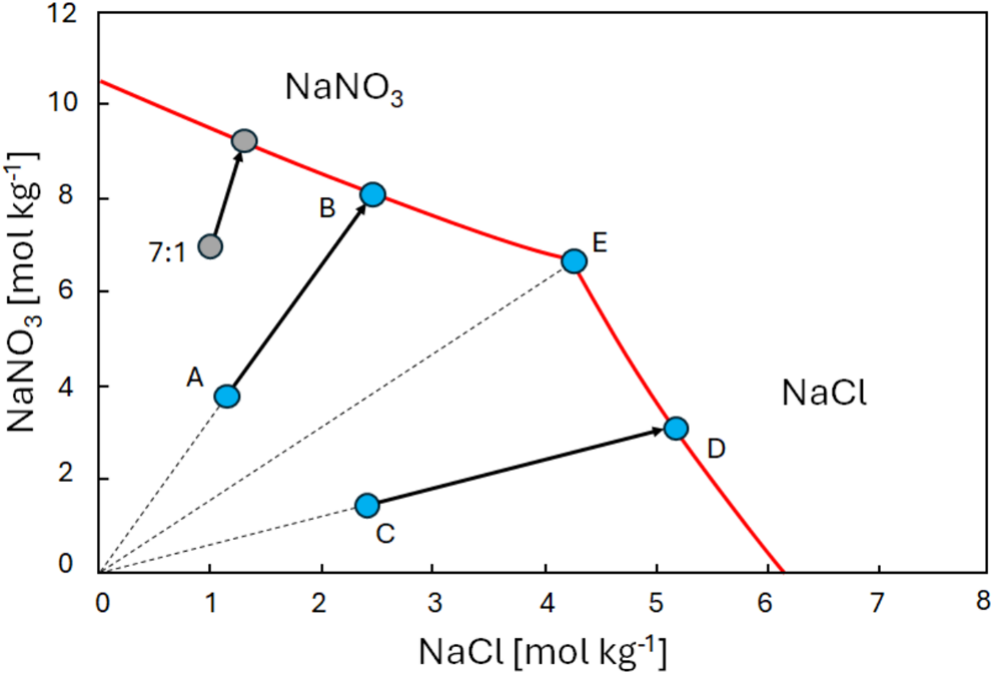
Ternary phase diagram of NaCl–NaNO_3_–H_2_O at 22 °C. The letters A–E refer to various crystallization paths that can be followed during a drying experiment, as discussed, whereas the ratio 7:1 is an example of the starting point of one of the experiments performed and discussed.

As an example, Figure 2 shows the ternary phase diagram of the system chosen in this study, i.e., NaCl–NaNO_3_–H_2_O at 22 °C, which indicates the solubilities of NaCl and NaNO_3_ in a mixed solution (see also ref (15)). For example, from this diagram, we can observe that the solubility of both salts reduces as the concentration of the secondary salt, NaNO_3_, increases, e.g., due to drying as water is removed. The red solid line is the equilibrium solubility line. A liquid phase, i.e., a salt solution where salt ions are dissolved in water, exists below this line, and solid salt(s) and, depending on the situation, salt solution coexists above this line, i.e., NaCl/NaNO_3_ crystals and salt solution. The diagram also reveals the crystallization pathways of mixed solutions considering different concentrations, i.e., when water evaporates during drying. For example, the concentration increases (water evaporates from a solution) when the initial composition (indicated as A) follows line AB, and the ratio between the salts will remain constant. At point B, the solution reaches saturation with respect to NaNO_3_, which starts to crystallize. The crystallization of NaNO_3_ causes a decrease of the sodium and nitrate content in the solution, followed by an increase of sodium and chloride concentration from B to E, i.e., the ratio of the salts will change from B to E. At E, saturation with respect to NaCl is reached, which will also start to crystallize out, and the ratio between the salts will remain constant. Vice versa, starting from point C, the solution first reaches saturation with respect to NaCl (at point D) changing the solution properties moving from D to E until NaNO_3_ crystallizes (at point E). In the given diagram, these processes are not dynamic and exclude kinetic influences, i.e., this was determined under semi-equilibrium conditions.

The goal of this study is to assess the accuracy of the equilibrium theory and evaluate its applicability when drying a porous medium saturated with a salt mixture where there is no longer an equilibrium condition, i.e., when advection is dominant. To this end, we have conducted a series of experiments to measure the ratio of NaCl to NaNO_3_ during the drying process. As we cannot observe crystallization directly in a porous material, we have used NMR to measure the ratio of the salt mixture, thereby gaining direct insights into the ongoing crystallization process. To achieve this, we used a specialized 4.7 T NMR setup, which will be described first, followed by a discussion on the experimental results. Finally, we will discuss the implications of these findings for our understanding of salt mixture crystallization in porous media during drying.

## 2 Experimental Setup

### 2.1 NMR Basics

A spin–echo is a phenomenon that occurs when nuclei, such as ^1^H, ^23^Na, and ^35^Cl, are exposed to a constant magnetic field and radio frequency (RF) pulses. These nuclei have a property called magnetic moment, which can be altered by the RF pulses. The nuclei will eventually return to their original state through two processes: spin–lattice (*T*_1_) relaxation and spin–spin (*T*_2_) relaxation. The strength of the spin–echo signal depends on these relaxation mechanisms and is given by^15,16^
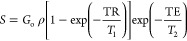
3where *G*_0_ is the absolute sensitivity (*G*_0_ = 1 for ^1^H, 0.0925 for ^23^Na, and 0.0035 for ^35^Cl), ρ is the density, *T*_1_ is the spin–lattice or longitudinal relaxation time, TR is the repetition time of the spin echo experiment, *T*_2_ is the spin–spin or transverse relaxation time, and TE is the so-called spin–echo time.^17^

As can be seen, the sensitivity is low for ^35^Cl, and as the signal-to-noise ratio is proportional to the magnetic field B^(3/2)^, these experiments were performed at a magnetic field of 4.7 T. Both ^23^Na and ^35^Cl have a spin of 3/2 and hence have a quadrupole moment. However, for the materials used in this study, i.e., Bentheimer sandstone, it is seen that we are in the so-called fast diffusion regime, and as a result, the movement and the tumbling of the ions are fast compared to the time scale of the NMR experiment. Therefore, the ions do not feel a net electrical field gradient (EFG) [see, refs (18,19)]. Hence, no splitting occurs, and a monoexponential relaxation behavior is observed. As a result, we again find a linear calibration for both the ^23^Na and ^35^Cl signals as a function of the concentration.

Therefore, the signal of ^35^Cl in the mixture of NaCl-NaNO_3_ will represent the concentration of the NaCl in the mixture. On the other hand, the total signal of the ^23^Na will represent the total concentration of NaCl-NaNO_3_ present. Hence, by subtracting the concentration of NaCl from the total signal NaCl-NaNO_3_ present, we can obtain the NaNO_3_ concentration.

### 2.2 NMR Setup

**Figure 3:**
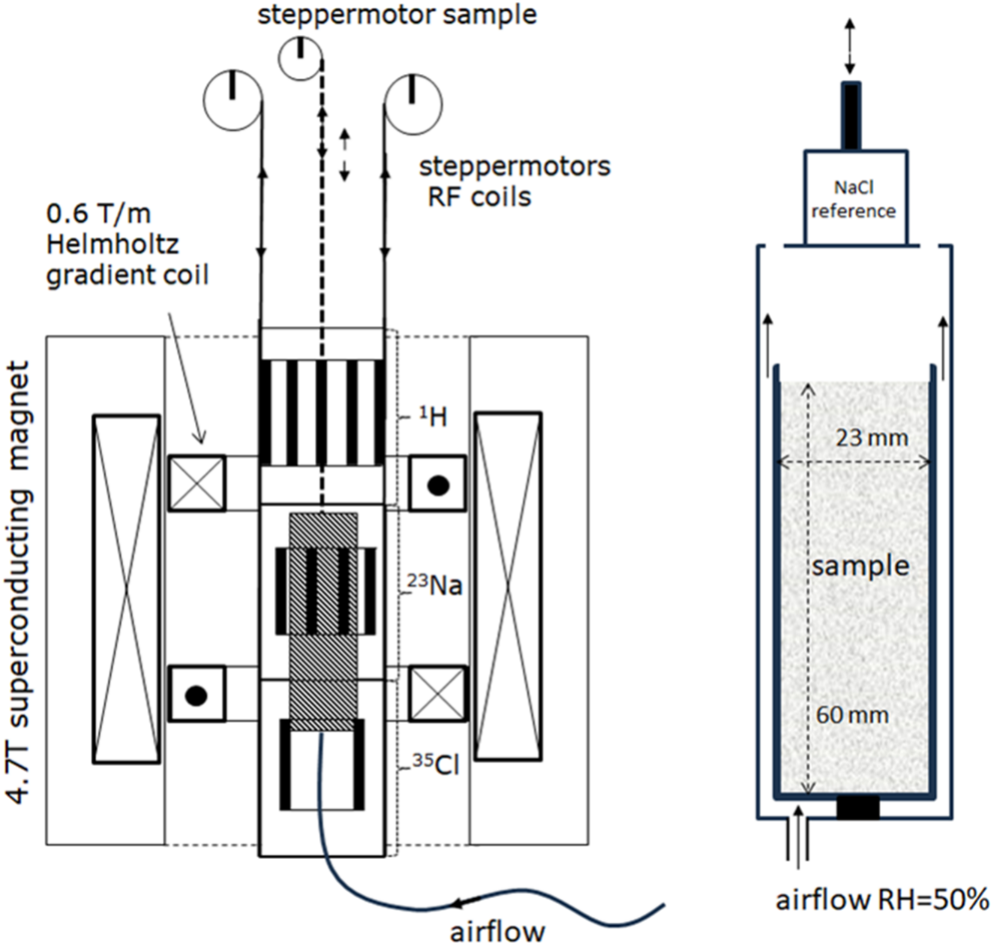
Schematic of the NMR setup for measuring ^1^H, ^23^Na, and ^35^Cl signal during drying of the sample saturated with a salt mixture solution. The main field is provided by a 4.7 T superconducting magnet, which is equipped with an anti-Helmholtz gradient coil set, providing a maximum gradient of 0.6 T/m. Using three stepper motors, the RF coil of interest can be moved toward the center of the gradient coils. The sample can be moved through the setup using a separate stepper motor.

In this study, we use a specially designed NMR setup to measure the concentrations in a solution, and more detailed information on the setup can be found in refs (20,21) In Figure 3, a schematic diagram is given for the setup. This setup uses 3 separate RF coils, which can be selected, resulting in the optimum sensitivity for all nuclei measured in this study. Using a separate stepper motor, the sample can be moved through the setup, and hence, one can measure the concentration distribution of both NaCl and NaNO_3_ over the sample. The samples used are Bentheimer cylinders of 23 mm diameter and a length of 60 mm. In order to induce drying, air with a RH of 50% is blown over the top of the sample. With the spin–echo times used in this study, i.e., TE = 300 μs, no signal is obtained from crystals. For measuring the signals of ^23^Na and ^35^Cl, a Carr–Purcell–Meiboom–Gill (CPMG) sequence was used with an echo time of 300 μs, and in total, 8 echoes were acquired, over which the average is taken. All measurements were performed at room conditions, i.e., 22 °C.

## 3 Experimental Results

### 3.1 Measured Profiles

**Figure 4:**
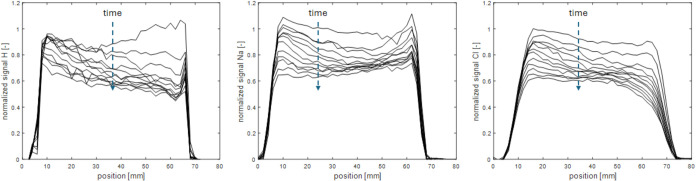
Measured normalized moisture H (left), Na (middle), and Cl (right) profiles for a sample initially saturated with a solution of a mixture of 3 m NaCl and 3 m NaNO_3_, i.e., a ratio of 1:1. The profiles are given every day, for a total of 14 days. Here, the Na profile represents the total Na signal, hence reflecting both the NaCl and NaNO_3_, whereas the Cl signal only reflects the NaCl content. With the chosen NMR parameters, no signal is obtained from the crystals.

Drying experiments were performed using Bentheimer sandstone samples, and a schematic diagram of the drying setup is given in Figure 3. This Bentheimer sandstone is widely used due to its simple mineralogy and the quite homogeneous and well-connected pore space, and it has a porosity of 0.24 and a dominant pore size of 11 μm.^22^ The samples were initially capillary saturated with various ratios for the concentration of the mixture of NaCl-NaNO_3_, i.e., 3 m:0 m, 3 m:3 m, 2.05 m:3 m, 2 m:5 m, 1 m:7 m, and 0 m:7 m. That is from a pure NaCl solution over a mixture of NaCl-NaNO_3_ solutions to a pure NaNO_3_ solution. As an example, Figure 4 shows the daily profiles measured over 14 days of moisture content (based on H signal), Na signal, and Cl signal during the drying of a 3 m:3 m solution.

As shown in the moisture profiles, a decrease is seen, indicating the sample is drying, but no drying front is observed within the 14 days of this experiment, i.e., the top of the sample does not get dry. This shows that the drying is externally limited; specifically, drying is limited by the external drying conditions in this experiment (see, ref (23)). Indeed, in all experiments reported in this study, no drying front was observed during the entire measured period, which generally lasted 14 days. Based on the drying curve, i.e., total moisture content as a function of time, the Peclet number was estimated, and for all experiments performed in this study, the Peclet number is in the range from 5 up to 10, indicating advection is dominant in these drying experiments.

The measured Cl signal profiles reflect the NaCl profiles. Hence, the decrease near the drying surface of the Cl signal can be directly linked to a decrease of NaCl. The interpretation of the measured profile for Na is however not straightforward as the measured Na signal profile is a combination of the signal of both NaCl and NaNO_3_.

### 3.2 Measured Salt Concentration Ratios as a Function of Time

As the one-dimensional NMR resolution for H, Na, and Cl are different and since we want to compare various drying measurements with different ratios, we have chosen to represent the drying experiments in a different form as to be able to interpret the data directly (similar to an efflorescence pathway diagram^24^). Since the NMR only measures free ions within the porous medium (i.e., it does not detect ions incorporated into crystals), we can represent this drying experiment in terms of the total free NaNO_3_ content plotted against the total free NaCl content, denoted as *cθ*. Here, *c* is the concentration of the salt, and θ is the moisture content, so their product *cθ* reflects the total free ion content. (see, refs (24,25)). During a drying experiment, the NaCl and/or NaNO_3_ content will decrease due to crystallization, and hence, the ratio will change (as discussed in the Section 1). Hence, by looking at the ratio of the total amount of the still soluble salt of NaCl and NaNO_3_, we can distinguish which salt is crystallizing in the Bentheimer sandstone indirectly.^26^

**Figure 5:**
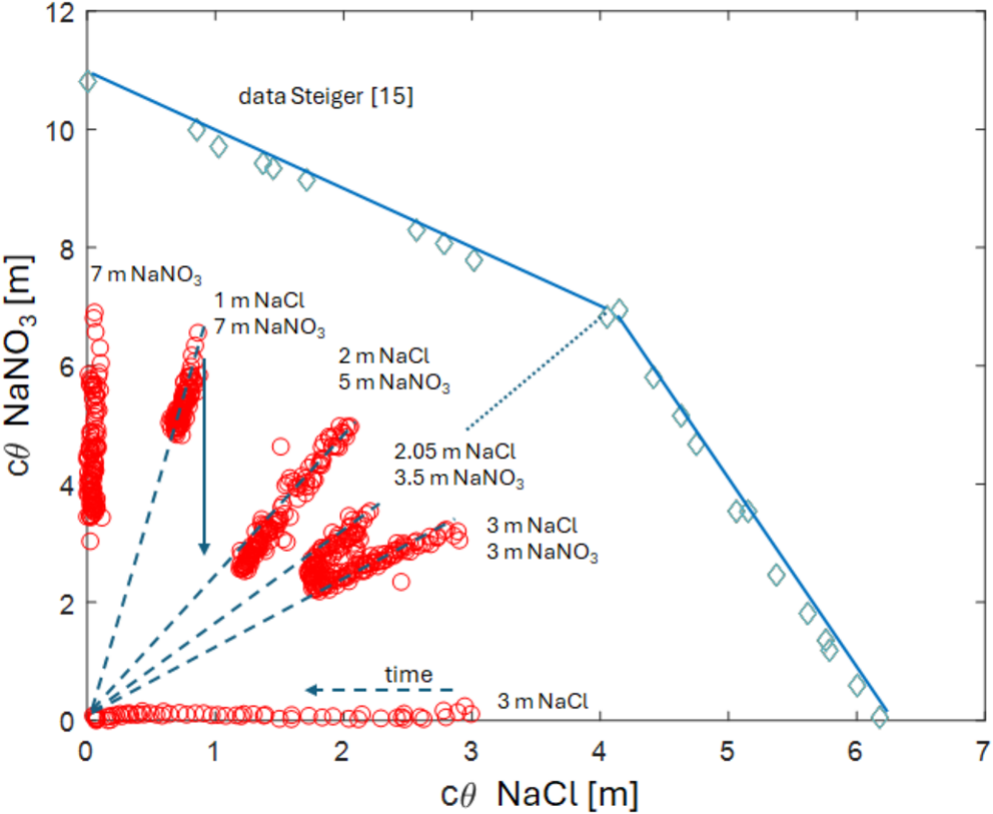
Total amount of free NaNO_3_ as a function of the total amount of free NaCl as determined from the measured ion concentration profiles during the drying experiments as a function of time at 22 °C. The total amount of free ions was determined every 3 h for at least 14 days for every drying experiment.

In Figure 5, we have plotted the results for all drying experiments for all profiles measured, specifically, the total amount of free NaNO_3_ content is plotted as a function of total amount of free NaCl. As can be seen in both the pure NaCl and NaNO_3_ solutions, once the drying begins, their content decreases as crystals form near the surface, resulting in a decrease in total free ion content over time, as expected, due to crystallization.

As an example of a mixture, we can look at the 1:7 ratio experiment. According to the equilibrium phase diagram in Figure 2, as soon as drying starts, the concentration of the mixture will increase until it reaches the equilibrium solubility line for NaNO_3_. In other words, the initial ratio remains constant because neither the total NaCl nor the NaNO_3_ content decreases at this stage. As drying continues, NaNO_3_ will start to crystallize out from the mixture, which reduces the free NaNO_3_ content. At the same time, the NaCl concentration will increase, leaving the total free NaCl content unchanged. Consequently, only the free NaNO_3_ content declines while the free NaCl content stays constant, as indicated by the arrow in Figure 5. This implies that the ratio between the salts in the Bentheimer sandstone will change during the crystallization. However, the experimental results show that the ratio of the salt mixture during this experiment remains constant. Indeed, for all experiments performed in this study (where *Pe* > 1), the drying experiments indicate that the concentration ratio remains constant from the onset of the drying. This is in sharp contrast with the equilibrium theory, as presented in the Section 1. Hence, these experiments demonstrate that the equilibrium approximation, as explained in the Section 1, is no longer valid under the conditions of these drying experiments where *Pe* > 1.

### 3.3 Analysis of Measured Ratios as a Function of Time

**Figure 6:**
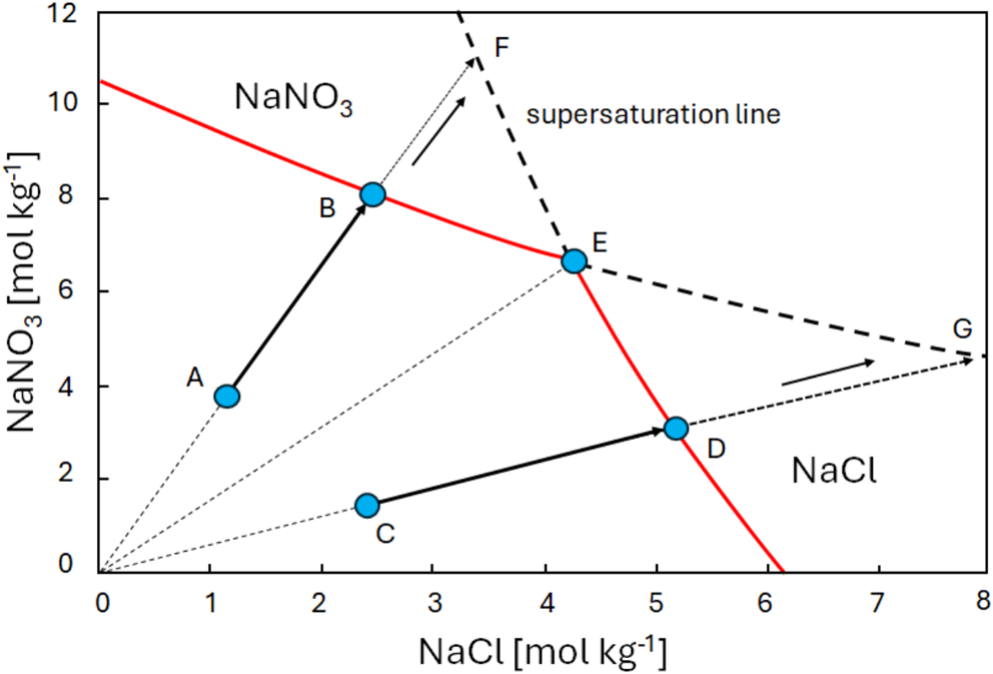
Ternary phase diagram of NaCl–NaNO_3_–H_2_O at 22 °C.^4^ The dashed lines (continuing the solid red lines) show the extension of the supersaturation line for NaCl and NaNO_3_.

For a possible explanation of the experiments presented in Figure 5, the ternary phase diagram of NaCl–NaNO_3_–H_2_O is revisited and is given in Figure 6. Here, the saturation lines for both NaCl and NaNO_3_ have been extended (dashed lines) in order to consider supersaturation. Again, as drying begins, water evaporates from the solution with an initial composition at point A, causing the solution to become more concentrated and follow line AB, thus maintaining a constant ratio, as seen in the experiments. At point B, the solution reaches saturation with respect to NaNO_3_. However, from the drying experiment, we observe that the ratio stays constant throughout the experiment. This would indicate that the supersaturation of NaNO_3_ increases until the supersaturation point for NaCl is reached at point F. Once the salt mixture reaches point F, NaCl begins to crystallize, and these crystals can act as nucleation sites for NaNO_3_. If crystallization occurs rapidly enough, the ratio of NaCl to NaNO_3_ will be governed completely by the transport of ions toward the surface, i.e., by the Peclet number (see, refs (12−14)). As a result, the concentration ratio of the salt mix remains constant during the drying process, as observed in these experiments. Here, one should keep in mind that, locally, there can be a distortion of the ratio; however, as the measured overall ratio stays constant, this indicates the crystallization rate of both salts is driven by the advection process (*Pe* > 1). Consequently, as a salt mixture with a certain ratio is transported by advection toward the surface, the ratio remains constant because both salts crystallize near the surface at the same rate at which they arrive at the surface.

In the case of starting at point C, again initially during the drying, the ratio will be constant until reaching point D. From this point on, the experiment seems to indicate that in this case, the solution supersaturates with respect to NaCl until reaching the supersaturation line at point G. From this moment on, NaNO_3_ will start to crystallize and act as a nucleation site for NaCl and, as a result, also in this case, the ratio will remain constant during drying.

It is important to note that this is a conceptual model (as given in Figure 6) envisioned to explain the experimental results presented. Supersaturation will only occur in a thin layer near the surface for a limited time, and with the current NMR resolution, it is likely undetectable. For some salts, such as Na_2_SO_4_, supersaturation has been measured in porous media and can be inferred from the concentration measured in such media.^27,28^ However, it should be noted that for a salt like Na_2_SO_4_, a significant supersaturation can develop over that area, substantially contributing to the measured overall concentration.^27,28^ Since no change in the mixture ratio was observed in the Bentheimer sandstone, this suggests that if supersaturation occurs, it is confined to a very small area and does not affect the overall salt mixture ratio.

## 4 Conclusions

Using NMR, one can measure the ratio of a salt mixture solution during drying. The measurements in this study show that the equilibrium phase diagram alone is not enough to describe the physical processes that occur in porous media drying experiments. In the case of forced drying, where advection of the ions is dominant (i.e., when the *Pe* > 1), the experiments suggest that we have to take supersaturation into account and that crystallization rate is driven by transport toward the surface. As a result, the ratio of a salt mixture will remain constant throughout the experiments, as was seen for the ternary system Na^+^, Cl^–^, NO_3_^–^, resulting in the formation of both NaCl and NaNO_3_. The experiments indicate that the rate of evaporation, in combination with the effect of supersaturation and solution transport in the pore system, allows the saturation degree given by the phase diagram to be surpassed. This phenomenon is critical for the development of accurate transport models and for assessing mixed salt systems, and it should be considered when relying solely on phase diagrams.

As this study shows, the Peclet number and the associated drying conditions are important factors in understanding the crystallization behavior of a mixture, with practical implications for the study of damage to cultural heritage objects and broader geoscientific contexts. For example, in cultural heritage research, salts are often analyzed by scraping them from the surface (to avoid damaging the object). However, if we understand that the drying conditions, such as exposure to the outside environment, result in a *Pe* > 1, we can infer that the salt mixture on the surface will be representative of the mixture inside the object. On the other hand, if the object is in an indoor environment where *Pe* ≤ 1 (i.e., representing more equilibrium conditions), the salt mixture at the surface may initially reflect the internal composition, but as drying progresses (as discussed with the 7:1 ratio example), the ratio of salts crystallizing on/near the surface will change. As a result, the salt ratio measured at the surface will no longer represent the ratio inside the object. Thus, caution should be exercised when interpreting salt ratio measurements that rely only on the salts found at the surface.
